# Metagenome-assembled genomes from *Spartina alterniflora* salt marsh roots and sediments along the U.S. Atlantic coast

**DOI:** 10.1128/mra.00791-26

**Published:** 2026-08-25

**Authors:** Jose L. Rolando, Gabrielle M. Krueger, Katherine Duchesneau, Joel E. Kostka

**Affiliations:** 1 Microbiology and Cell Science Department, Institute of Food and Agricultural Sciences (IFAS), Fort Lauderdale Research and Education Center, University of Florida 3463, Davie, Florida, USA; 2 School of Biological Sciences, Georgia Institute of Technology 1372, Atlanta, Georgia, USA; 3 School of Integrative Plant Science, Cornell University 5922, Ithaca, New York, USA; 4 School of Earth and Atmospheric Sciences, Georgia Institute of Technology 1372, Atlanta, Georgia, USA; 5 Center for Microbial Dynamics and Infection, Georgia Institute of Technology 1372, Atlanta, Georgia, USA; University of Manitoba, Winnipeg, Manitoba, Canada

**Keywords:** salt marsh, root microbiome, sediment microbiome, sulfate-reducing bacteria, sulfur-oxidizing bacteria, metagenomics

## Abstract

*Spartina alterniflora* is the dominant plant in low-elevation salt marshes along the U.S. Atlantic coast. Here, we present 434 metagenome-assembled genomes assembled from 36 metagenomic libraries of *S. alterniflora* sediment and root samples in Georgia, Virginia, and Massachusetts. Samples were collected across natural environmental stress gradients at all sites.

## ANNOUNCEMENT

Salt marshes are intertidal wetlands located at the land-sea interface. In the United States, low-elevation salt marshes are dominated by the smooth cordgrass *Spartina alterniflora*. The *S. alterniflora* root-associated microbiome is dominated by microorganisms that mediate transformations in the sulfur cycle (1–3). Furthermore, microbiome assembly in salt marshes is shaped by natural stress gradients formed by sharp differences in sediment redox potential, sulfide concentration, oxygen availability, and salinity (2, 4). However, despite their role in global biogeochemical cycles, plant-associated microbiomes in coastal wetlands remain understudied compared to those from agricultural and model systems (1, 5–7).

**Fig 1:**
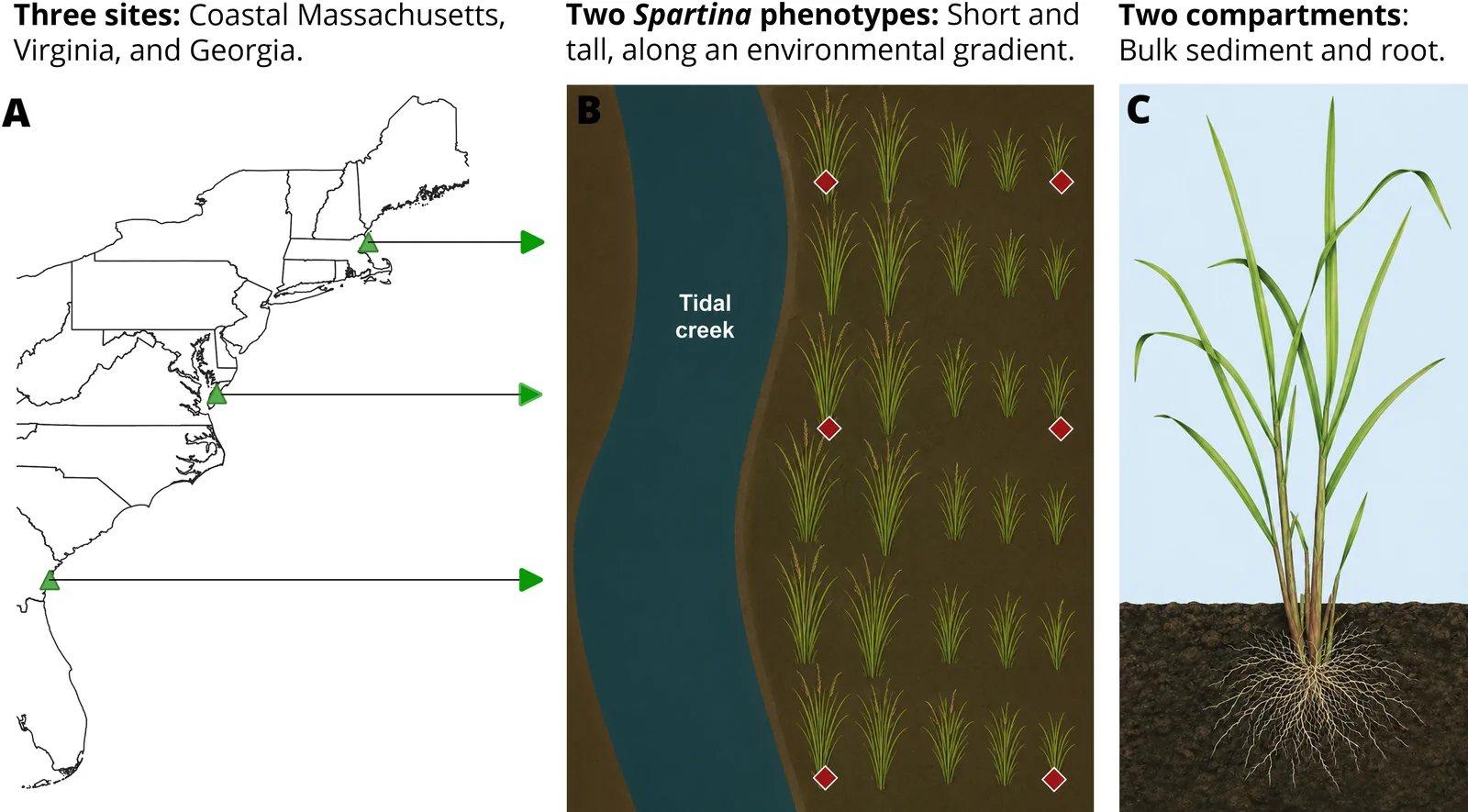
Sampling design along multiple gradients. Metagenomes were generated from three NSF-LTER sites spanning the U.S. Atlantic coast (**A**): Plum Island Ecosystems (Massachusetts), Virginia Coast Reserve (Virginia), and Georgia Coastal Ecosystems (Sapelo Island, Georgia). At each site, samples were collected from the two *Spartina alterniflora* phenotypes (**B**): the tall form at the creek bank, which is flushed frequently by tidal exchange, and a short form in the marsh interior, where more stagnant, waterlogged conditions impose stronger sulfide, anoxia, and salinity stress. For each sampling point, two microbiome compartments were sampled (**C**): bulk sediment and roots, with three biological replicates per compartment, phenotype, and site. Panels **B** and **C** were modified using ChatGPT (GPT-5.5) with OpenAI’s integrated image generation capabilities.

Here, we report metagenomic sequencing and metagenome-assembled genomes (MAGs) retrieved from sediment and root samples collected along environmental stress gradients at three Long-Term Ecological Research (LTER) sites on the U.S. Atlantic coast: Georgia Coastal Ecosystems on Sapelo Island, GA; Virginia Coast Reserve, VA; and Plum Island Ecosystems, MA (Fig. 1). At each site, we collected three biological replicates per microbiome compartment (bulk sediment and root) from the tall and short forms of *S. alterniflora*. Compartment separation was performed as described by Rolando et al. (2). DNA was extracted from 0.25 g of initial material using the Qiagen DNeasy PowerSoil Pro Kit following the manufacturer’s instructions. Metagenomic sequencing was performed at the Joint Genome Institute (JGI) on an Illumina NovaSeq X instrument (150 bp PE, Illumina Regular Fragment, 400 bp insert size), yielding a total of 850 Gbp (5.6 billion reads). Metagenomic reads were assessed for quality with FastQC (8), and quality filtered using BBTools (9) (versions and parameters for all bioinformatic tools are listed in Table 1). Assemblies were generated on corrected reads with bbcms (9), using metaSPAdes (10). Genomes were recovered using the automated metagenome-binning tool SemiBin2 v2.1.0 (11), with a 3,000 bp minimum contig cutoff. Genomes with a completeness of less than 50% or contamination greater than 5% were discarded based on CheckM2 (12), resulting in 434 MAGs. Recovered MAGs were dereplicated into 201 genomospecies by clustering at 95% average nucleotide identity with dRep (13, 14). Taxonomic assignment was performed with GTDB-Tk (15) using the GTDB database, Release 11-RS232 (16). Genome annotation was performed with NCBI’s Prokaryotic Genome Annotation Pipeline (PGAP) (17).

**TABLE 1:** Bioinformatic tools, versions, and parameters used for metagenome quality control, assembly, and MAG recovery

Process	Software	Version	Parameters
Read quality control	FastQC	v0.12.1	default
Read quality filtering	RQCFilter2 (BBTools)	v39.03	barcodefilter=f clumpify=t ddist=50 dedupe kapa=t khist=t maq=3 maxns=3 minlen=51 mlf=0.33 opticaldupes phix=t pigz=t qtrim=r removecat=t removedog=t removehuman=t removemicrobes=t removemouse=t rna=f trimfragadapter=t trimpolyg=5 trimq=0
Read correction	bbcms (BBTools)	v38.90	mincount=2 highcountfraction=0.6
Contig mapping	bbmap (BBTools)	v38.86	build=1 fastareadlen=500 ambiguous=random
Assembly	metaSPAdes	v3.15.2	-m 2000 --only-assembler -k 33,55,77,99,127 --meta
Binning	SemiBin2	v2.1.0	--self-supervised
MAG quality control	CheckM2	v1.1.0	default
MAG dereplication	dRep	v3.4.2	default
Taxonomic assignment	GTDB-Tk	v2.7.1	classify_wf
MAG annotation	PGAP	v6.11	default

Of the 201 dereplicated MAGs, 189 were classified as bacteria and 12 as archaea. The taxonomic breadth of our data set includes MAGs from 26 phyla, including methanogenic and ammonia-oxidizing archaea from the genera *Methanolacinia* and *Nitrosopumilus*, respectively. At the phylum level, the most prevalent bacterial taxa were Pseudomonadota (59 MAGs total, 53 in the class Gammaproteobacteria) and Desulfobacterota (52 MAGs total, 30 in the Desulfobacterales). This data set contains *S. alterniflora* root symbionts closely related to chemosymbionts of marine invertebrates, including seven dereplicated *Candidatus* Thiodiazotropha MAGs, recovered from all sites along the latitudinal gradient. *Ca*. Thiodiazotropha has been implicated as a key player in nitrogen fixation in the root zone of *S. alterniflora* in Georgia salt marshes (1). These genomic resources augment our capacity to study the root and sediment microbiomes of coastal wetlands across latitudinal, stress, and root-proximity gradients.

## Data Availability

Raw metagenomic and metagenome-assembled genomes have been deposited in the BioProject database (http://ncbi.nlm.nih.gov/bioproject) under accession code PRJNA1476290. GenBank accession numbers for all metagenome-assembled genomes are shown in Table S1 in a Zenodo repository (18).
